# Astaxanthin prevents pulmonary fibrosis by promoting myofibroblast apoptosis dependent on Drp1-mediated mitochondrial fission

**DOI:** 10.1111/jcmm.12609

**Published:** 2015-06-27

**Authors:** Jinjin Zhang, Pan Xu, Youlei Wang, Meirong Wang, Hongbo Li, Shengcui Lin, Cuiping Mao, Bingsi Wang, Xiaodong Song, Changjun Lv

**Affiliations:** a Medicine Research Center, Binzhou Medical UniversityYantai, China; b Department of Respiratory Medicine, Affiliated Hospital to Binzhou Medical UniversityBinzhou, China; c School of Special Education, Binzhou Medical UniversityYantai, China; d Clinical Laboratory, Affiliated Hospital to Binzhou Medical UniversityYantai, China; e Department of Respiratory Medicine, Affiliated Hospital to Binzhou Medical UniversityYantai, China

**Keywords:** astaxanthin, pulmonary fibrosis, myofibroblast apoptosis, mitochondrial fission

## Abstract

Promotion of myofibroblast apoptosis is a potential therapeutic strategy for pulmonary fibrosis. This study investigated the antifibrotic effect of astaxanthin on the promotion of myofibroblast apoptosis based on dynamin-related protein-1 (Drp1)-mediated mitochondrial fission *in vivo* and *in vitro*. Results showed that astaxanthin can inhibit lung parenchymal distortion and collagen deposition, as well as promote myofibroblast apoptosis. Astaxanthin demonstrated pro-apoptotic function in myofibroblasts by contributing to mitochondrial fission, thereby leading to apoptosis by increasing the Drp1 expression and enhancing Drp1 translocation into the mitochondria. Two specific siRNAs were used to demonstrate that Drp1 is necessary to promote astaxanthin-induced mitochondrial fission and apoptosis in myofibroblasts. Drp1-associated genes, such as Bcl-2-associated X protein, cytochrome c, tumour suppressor gene p53 and p53-up-regulated modulator of apoptosis, were highly up-regulated in the astaxanthin group compared with those in the sham group. This study revealed that astaxanthin can prevent pulmonary fibrosis by promoting myofibroblast apoptosis through a Drp1-dependent molecular pathway. Furthermore, astaxanthin provides a potential therapeutic value in pulmonary fibrosis treatment.

## Introduction

Myofibroblasts contribute to the pathogenesis in pulmonary fibrosis because of their increased production of extracellular matrix (ECM) and fibrogenic mediators, resulting in the loss of alveolar function 1. The presence, differentiation and persistence of myofibroblasts determine the degree of fibrogenesis 2. Given that excessive myofibroblasts are eliminated by apoptosis in the normal wound repair process 3,4, the induction of myofibroblast apoptosis is a potential therapeutic strategy for both the prevention and treatment of pulmonary fibrosis.

Mitochondria are highly dynamic organelles that undergo constant fusion and fission; the resulting morphological changes in the mitochondrial network are necessary for the mitochondrial functions in living cells to adapt to various conditions 5,6. This phenomenon highlights the importance of homoeostasis between fission and fusion. Mounting experiments have shown that abnormal mitochondrial fission occurs during pathophysiological events, such as neurodegeneration, cellular aging and apoptosis 7,8. Dynamin-related protein-1 (Drp1), also called Dnm1 in yeast, belongs to the dynamin GTPase superfamily, which mediates the formation of a helical ring around the outer mitochondrial membrane to segment the mitochondria into discrete compartments 9,10. Previous research revealed that the inhibition of Drp1 by overexpression of Drp1K38A, a Drp1-dominant-negative mutant, reduced the mitochondrial fragmentation; extensively fragmented mitochondrial morphology was also associated with Drp1 translocation to the mitochondria 11,12. Therefore, regulation of Drp1-dependent mitochondrial fission is crucial for the development of drugs for intervening myofibroblastic apoptosis.

Astaxanthin, a non-provitamin A carotenoid, functions as a principal pigment in crustaceans, salmonoids and other organisms. Astaxanthin also demonstrates great applications in food, feed, nutraceutical and pharmaceutical industries 13,14. The European Commission considers natural astaxanthin as a functional food agent for preventing or reducing various disorders in humans and animals, such as neurodegeneration, cardiovascular diseases, diabetes and cancer 15,16. Our previous work revealed the preventive function of astaxanthin in pulmonary fibrosis, as well as its effects on the transdifferentiation, proliferation and apoptosis of myofibroblasts 17; however, we did not investigate the mechanism of astaxanthin in promoting myofibroblast apoptosis. Therefore, our present study investigated the mechanism of astaxanthin in promoting myofibroblast apoptosis through the signalling pathway of Drp1-mediated mitochondrial fission.

## Materials and methods

### Human tissue samples

Patients were diagnosed with pulmonary fibrosis through combined clinical, radiological and pathological findings according to the American Thoracic Society/European Respiratory Society consensus criteria 18. Biopsy samples from six patients were routinely placed in formaldehyde or glutaraldehyde for surgical and pathological evaluation. Control samples were obtained from six patients with primary lung carcinoma who underwent thoracic surgery. The protocol was approved by the local ethics committee, and written informed consent with accompanying images was obtained from the patients for publication of this report.

### Animal model

A pulmonary fibrosis model was established by the instillation of the chemotherapeutic agent bleomycin. Briefly, Sprague–Dawley (SD) rats weighing 200 g were purchased from the Yantai Green Leaf Experimental Animal Center and randomly divided into three groups (with 15 rats for each group): sham group, bleomycin group and bleomycin + astaxanthin group. Pulmonary fibrosis in rats was induced by a single intratracheal instillation of 5 mg/kg bleomycin in 0.3 ml of saline, whereas the rats in the sham group received equal volume of saline. The rats in the bleomycin + astaxanthin group were administered daily with 2 mg/kg astaxanthin beginning on the 14th day after bleomycin injury (*i.e*. post-inflammatory fibrotic phase) for 7 or 14 days 19, whereas the rats in other groups were given water. Rats were killed on the 21st or 28th day. Portions of tissue samples were immediately frozen in liquid nitrogen for quantitative real-time PCR (qRT-PCR) and Western blot analysis, fixed in 4% formaldehyde for immunohistochemistry, and fixed in 2.5% glutaraldehyde for ultrastructural observation. All the rats in our experiments received the guiding principles of care in accordance with the National Institutes of Health Guide for the Care and Use of Laboratory Animals.

### Cell model

Human lung fibroblast MRC-5 and lung adenocarcinoma 549 (A549) cell lines were purchased from the Cell Bank of the Chinese Academy of Sciences (Shanghai, China). Both cell lines were maintained at 37°C in DMEM supplemented with 10% newborn calf serum under a humidified atmosphere of 5% CO_2_. The cells were divided into four groups: control group, transforming growth factor-beta 1 (TGF-β1) group, astaxanthin group and TGF-β1+ astaxanthin group (*n* = 6 per group). The cells were pre-treated with 5 ng/ml TGF-β1 (Invitrogen, Carlsbad, CA, USA) for 72 hrs for transdifferentiation of myofibroblasts then co-treated with or without astaxanthin (24 and 18 μg/ml for A549 and MRC-5 cells, respectively) for another 48 hrs.

### Histopathological examination

Lung tissues of all the groups were fixed with 4% *para*-formaldehyde and later embedded in paraffin. Transverse sections of 4 μm-thick slices were stained with hematoxylin and eosin (Sigma-Aldrich, St. Louis, MO, USA) and Masson’s trichrome (Maxin Company, Fuzhou, China). The percentage of lung fibrosis was evaluated by counting the number of pixels in the digital images corresponding to the stained collagen areas by using Adobe Photoshop CS3, as previously described 20,21.

### Transmission electron microscopy observation

Cell and tissue samples were initially fixed in fresh 2.5% glutaraldehyde for at least 4 hrs at 4°C, post-fixed in 1% osmium tetroxide for 1.5 hrs, dehydrated in a gradient ethanol series and infiltrated with Epon812. The samples were then embedded and cured at 37°C for 12 hrs, 45°C for 12 hrs and 60°C for 24 hrs. Ultrathin sections were cut using ultracut E ultramicrotome (Leica, Wetzlar, Germany) and stained with uranyl acetate and lead citrate before observation under the JEM-1400 transmission electron microscope (TEM; JEOL Ltd., Tokyo, Japan).

### Immunofluorescence analysis

The tissue sections and cells cultured on coverslips were fixed in 4% *para*-formaldehyde, rinsed with PBS, incubated in 0.5% Triton X-100 for 20 min. and blocked nonspecific antigens with 10% serum. Samples were then incubated overnight at 4°C with primary antibodies including anti-α-smooth muscle actin (α-SMA), anti-Bcl-2-associated X protein (Bax), anti-Drp1, anti-p53-up-regulated modulator of apoptosis (PUMA), anti-surfactant-associated protein (SP-C) and anti-Snail (Santa Cruz Biotechnology, Watsonville, CA, USA). Subsequently, treated samples were rinsed with PBS and then incubated with secondary antibodies labelled with FITC (fluorescein isothiocyanate) and Cy3 for 60 min. at 37°C, followed by incubation with Hoechst 33258 dye. Serums of the same species as the primary antibodies were used as the negative control. Cover slips or tissue sections were mounted on neutral glycerin and photographed under a confocal laser fluorescence microscope (Leica) with LAS AF software. The mean fluorescence intensity was expressed in fluorescence units, and dual-positive cells were counted in six visual fields that were randomly selected from each slide by two blinded observers.

### *In situ* terminal deoxynucleotidyl transferase dUTP nick end labelling

Terminal deoxynucleotidyl transferase dUTP nick end labelling (TUNEL) assay was performed in the paraffin sections of the lung tissues by using the Fluorometric TUNEL System from KeyGen Biotech in accordance with the manufacturer’s instructions. Sections were counterstained with Hoechst 33258 dye for nuclear staining and photographed using a confocal laser fluorescence microscope (Leica). TUNEL-positive cells were counted in six visual fields that were randomly selected from each slide by two blinded observers.

### Flow cytometry measurement

Suspended and adherent cells were collected and washed with cold PBS. Up to 500 μl of binding buffer was added to resuspend the cells. Annexin V-FIFC (5 μl) and propidium iodide staining solution (5 μl; Beyotime, Jiangsu, China) were added for 20 min. and incubated in the dark. The apoptotic rate was measured by flow cytometry (Beckman, Fullerton, CA, USA).

### Mitochondrial staining

Cells were plated onto coverslips coated with 0.01% poly-l-lysine. Upon treatment following the protocol described previously, the cells were stained for 35 min. with 200 nM MitoTracker Red CMXRos (Molecular Probes; Invitrogen, Carlsbad, CA, USA), and immunofluorescence staining was performed. Samples were visualized using a laser scanning confocal microscope (Leica).

### Live cell imaging

Cells were plated onto 35 mm glass-bottom dishes (Corning, Corning, NY, USA) and allowed to adhere for 24 hrs. The cells were then treated with 5 ng/ml TGF-β1 (Invitrogen) for 72 hrs. After staining with 100 nM MitoTracker Red CMXRos (Molecular Probes) for 35 min., the cells were administered with or without astaxanthin, and cell imaging was performed. The cells were observed on a live cell imaging system (DV ELITE; API, Fairfield, CT, USA) with a temperature-controlled incubation chamber maintained at 37°C. MitoTracker Red CMXRos was excited with a Chameleon Multiphoton laser emitting 579 nm laser pulses. Emission was collected at 599 nm.

### qRT-PCR analysis

Total RNA was isolated from lung tissues and cells by using TRIzol reagent (Invitrogen) then twofold serially diluted in nuclease-free water. Up to 2 μg of RNA was used for first-strand cDNA synthesis. qRT-PCR analysis was performed with the Rotor-Gene 3000 Real-time PCR system with the following reaction profiles: pre-denaturation at 95°C for 30 sec. and PCR amplification for 40 cycles with 95°C for 15 sec. and 60°C for 25 sec. PCR was followed by a melt curve analysis to determine the reaction specificity. The relative gene expression was calculated using standard ΔΔCt methods by Rotor-Gene 6 Software. GAPDH RNA was quantified as a control to normalize differences in total RNA levels. The following primers were used to quantify the Drp1 levels: Human: Drp1-F 5′-AGAACCAACCACAGGCAACTG-3′; Drp1-R 5′-GGCTGGCATAATTGGAATGG-3′; Rat: Drp1-F 5′-ATTGAAGGAACGGCAAAGTACATT-3′; Drp1-R 5′-CAGATTCTAAGGTTCGCCCAAA-3′.

### Transfection of siRNA

Two siRNA sequences targeting human Drp1 gene were synthesized by Guangzhou RiboBio Co., Ltd, Development zone, Scientific town,GuangZhou, China. For transfection, cells were placed on six-well plates. Transfections were performed with Lipo2000 for 24 hrs as directed by the manufacturer (Invitrogen). The final siRNA concentration was 25 nM. The transfected samples were treated as described above.

### Western blot analysis

Proteins of lung tissues and cells were extracted, and protein concentration was quantified using the bicinchoninic acid protein assay kit (Beyotime). Protein samples were separated by 15% SDS-PAGE for 2 hrs, transferred onto a polyvinylidene difluoride membrane and blocked in 5% non-fat milk for 2 hrs. Blots were incubated overnight with the specific primary antibodies against α-SMA, Snail, Bax, Drp1, cytochrome c, p53 and PUMA (Santa Cruz Biotechnology) at 4°C, followed by the addition of species-specific horseradish peroxidase-conjugated secondary antibodies with IgG, and further incubation for 1 hr at room temperature. Immunoreactive bands were visualized using the ECL-assay kit (Beyotime). For detection of mitochondrial Drp1, mitochondrial protein was isolated from the lungs of rats by using a mitochondria isolation kit (Beyotime). The band’s density was analysed and quantified using a ChemiScope series and ChemiAnalysis software.

### Statistical analysis

All data were presented as mean ± SD. Unpaired Student’s *t*-test was used for experiments comparing two groups, whereas one-way anova with Student–Newman–Keuls *post hoc* test was applied for experiments comparing three or more groups. All analyses were performed with SPSS statistical package (version 11.0 for Windows; SPSS Inc., Chicago, IL, USA). A *P* < 0.05 was considered statistically significant.

## Results

### Emergence of myofibroblasts in pulmonary fibrosis

**Figure 1:**
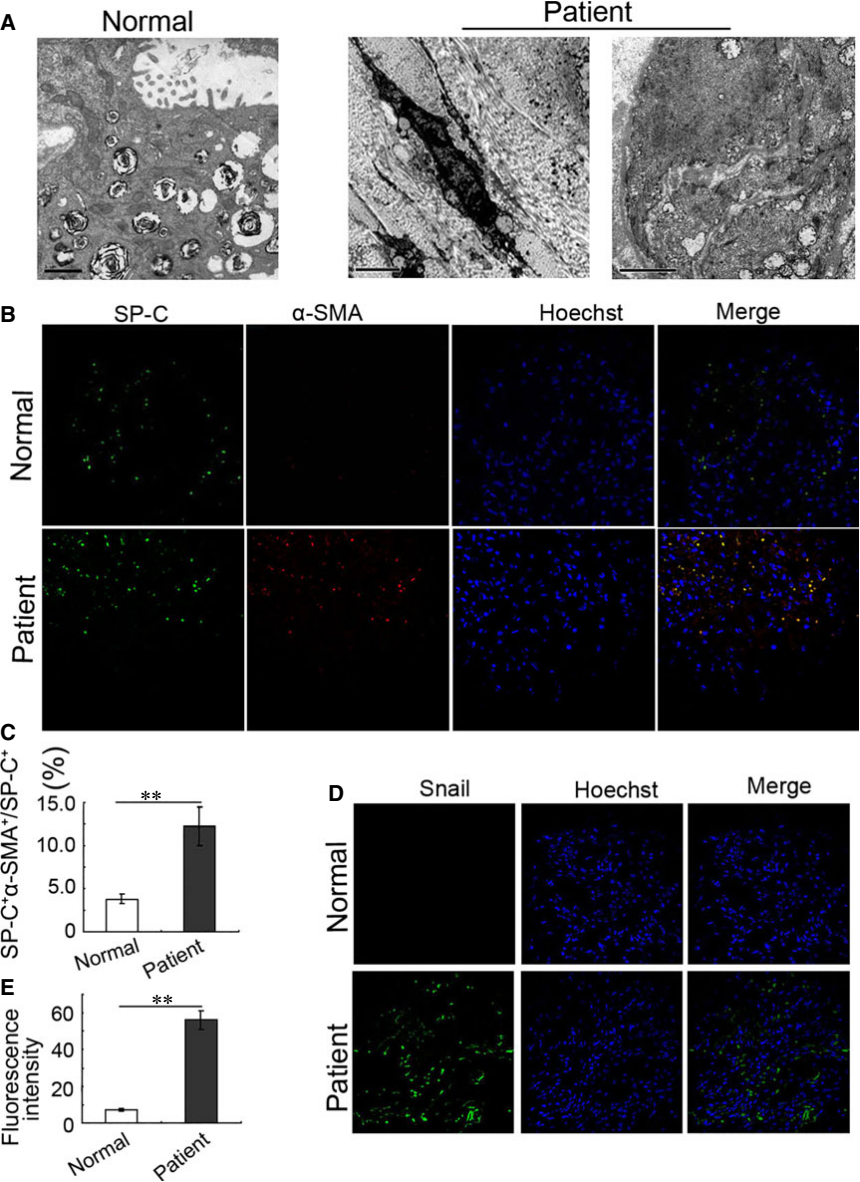
Emergence of myofibroblasts in patients with pulmonary fibrosis. (A) Under TEM, AECs-II contained many lamellar bodies with uniform density and ring-like arrangement in their cytoplasm and distinct microvilli on the surface in normal lung tissues (left panel). Myofibroblasts and collagen emerged in human fibrotic samples (middle panel). Bundles of actin microfilaments were visible in AECs-II (right panel). (B) Lung sections were stained with epithelial marker-SP-C and mesenchymal maker-α-SMA antibodies followed by incubation with fluorescent dye-tagged secondary antibodies. Hoechst was used to stain nuclei (blue). Yellow fluorescence represents fluorescence co-expression. SP-C (green), α-SMA (red). (C) The bar graph represents the number of dual-positive cells relative to SP-C^+^ cells. At least 200 SP-C^+^ cells per slide were counted. (D) The expression of transcription factor (Snail) increased in human fibrotic samples as detected by confocal immunofluorescence microscopy. (E) Bar graph represents fluorescence intensity of Snail. Data are expressed as mean ± SD, *n* = 6, ^**^P < 0.01 *versus* normal group, unpaired Student’s *t*-tests.

**Figure 2:**
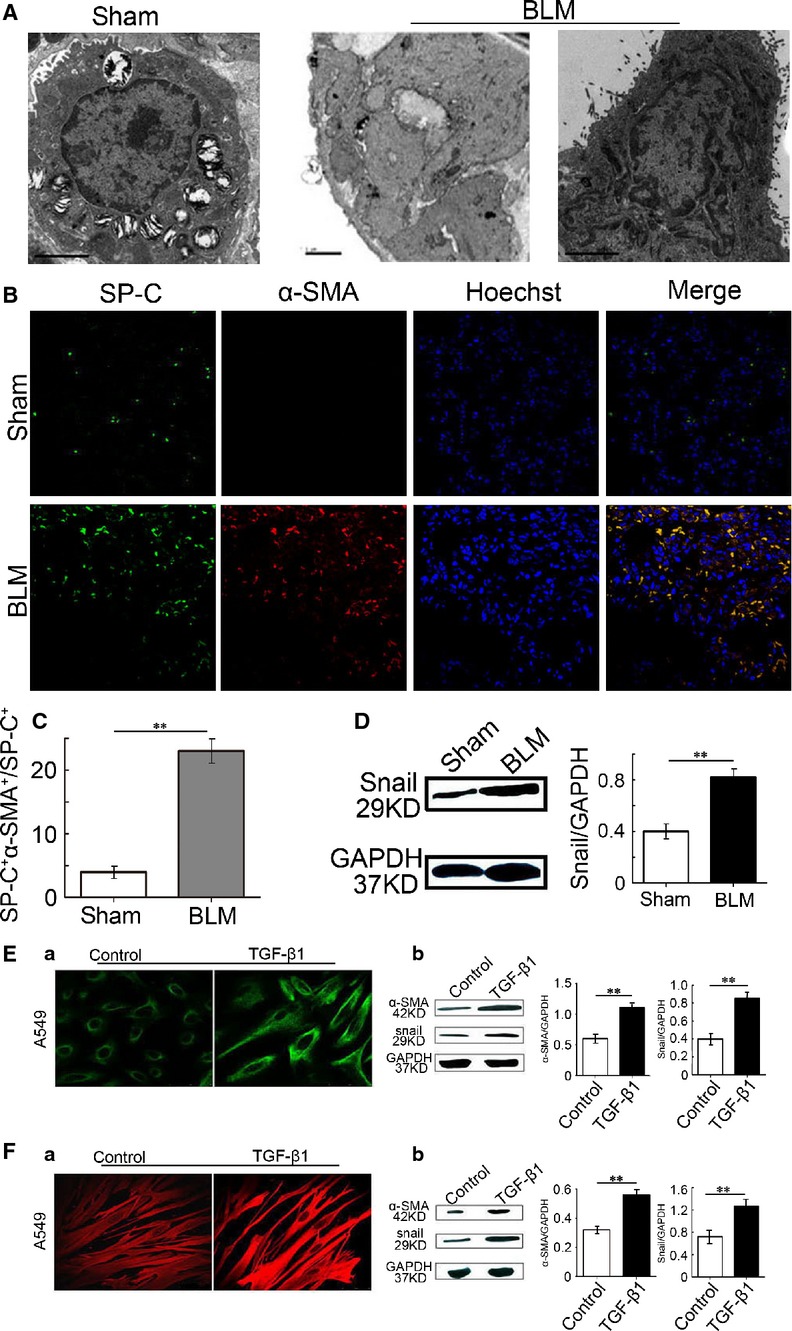
Emergence of myofibroblasts in animal and cell models. (A) Under TEM, AECs-II contained many lamellar bodies with uniform density and ring-like arrangement in their cytoplasm and distinct microvilli on the surface in the normal lung tissue (left panel). Emergence of myofibroblasts in rat fibrotic samples was detected by TEM (middle panel). AECs-II displayed cell–cell adhesion and bundles of intracellular actin in lung tissues from fibrotic rats (right panel). (B) The lung sections were stained with SP-C and α-SMA antibodies followed by incubation with fluorescent dye-tagged secondary antibodies. Hoechst was used to stain nuclei (blue). Yellow fluorescence represents fluorescence co-expression. SP-C (green), α-SMA (red). (C) Bar graph represents the number of dual-positive cells relative to SP-C-positive cells. At least 200 SP-C^+^ cells per slide were counted. (D) Western blot analysis showed the changes in Snail. (E) TGF-β1-induced activation of A549 cells (A) Actin stained with α-SMA in A549 cells (green). (B) Protein levels of α-SMA and Snail in A549 cells were detected by Western blot analysis. (F) TGF-β1-induced activation of MRC-5 cells (A) Actin stained with α-SMA (red) in MRC-5 cells. (B) Protein levels of α-SMA and Snail in MRC-5 cells were detected by Western blot analysis. Data are shown as mean ± SD, *n* = 6, **P < 0.01 *versus* sham group (C and D) or control group (E and F), measured by unpaired Student’s *t*-tests. BLM: bleomycin.

To examine the presence of myofibroblasts in human lung fibrosis, macroscopic and microscopic changes of autopsy samples from patients with pulmonary fibrosis were determined using TEM and laser scanning confocal microscope. The TEM results showed that myofibroblasts and excessive ECM existed in human fibrotic lung tissues (middle panel). Moreover, AECs-II contained bundles of filaments in its cytoplasm (right panel; Fig.1A). Simultaneously, SP-C^+^ (the AECs-II specific marker) and α-SMA^+^ (the myofibroblasts specific marker) dual-positive cells were significantly present in the human fibrotic lung tissues detected by immunofluorescence staining (Fig.1B and C). The levels of Snail, a well-established master transcription regulator of epithelial–mesenchymal transition during the development of pulmonary fibrosis, also increased in autopsy samples from patients with pulmonary fibrosis (Fig.1D and E). These findings confirmed that AECs-II can transdifferentiate into myofibroblasts in human pulmonary fibrosis. We also tested the emergence of myofibroblasts in bleomycin-induced rat and TGF-β1-stimulated cell fibrotic models by using the same methods. As shown in Figure2A, many myofibroblasts (middle panel) and AECs-II displaying mesenchymal phenotypes (right panel) were detected in rat fibrotic lung tissues. Moreover, the number of cells co-expressing SP-C and α-SMA and levels of Snail increased significantly in rat fibrotic lung tissues (Fig.2B–D). As the transition of myofibroblasts from their precursors was driven by TGF-β1, we stimulated MRC-5 and A549 cells with 5 ng/ml TGF-β1 for 72 hrs. The results also showed that α-SMA and Snail were significantly up-regulated in the TGF-β1 group (Fig.2E and F). Thus, the emergence of myofibroblasts, which can result from AECs-II *in vivo* and *in vitro*, was similar to that in actual patients. This finding indicated that our pulmonary fibrosis model was significant for this study.

### Astaxanthin prevented pulmonary fibrosis

**Figure 3:**
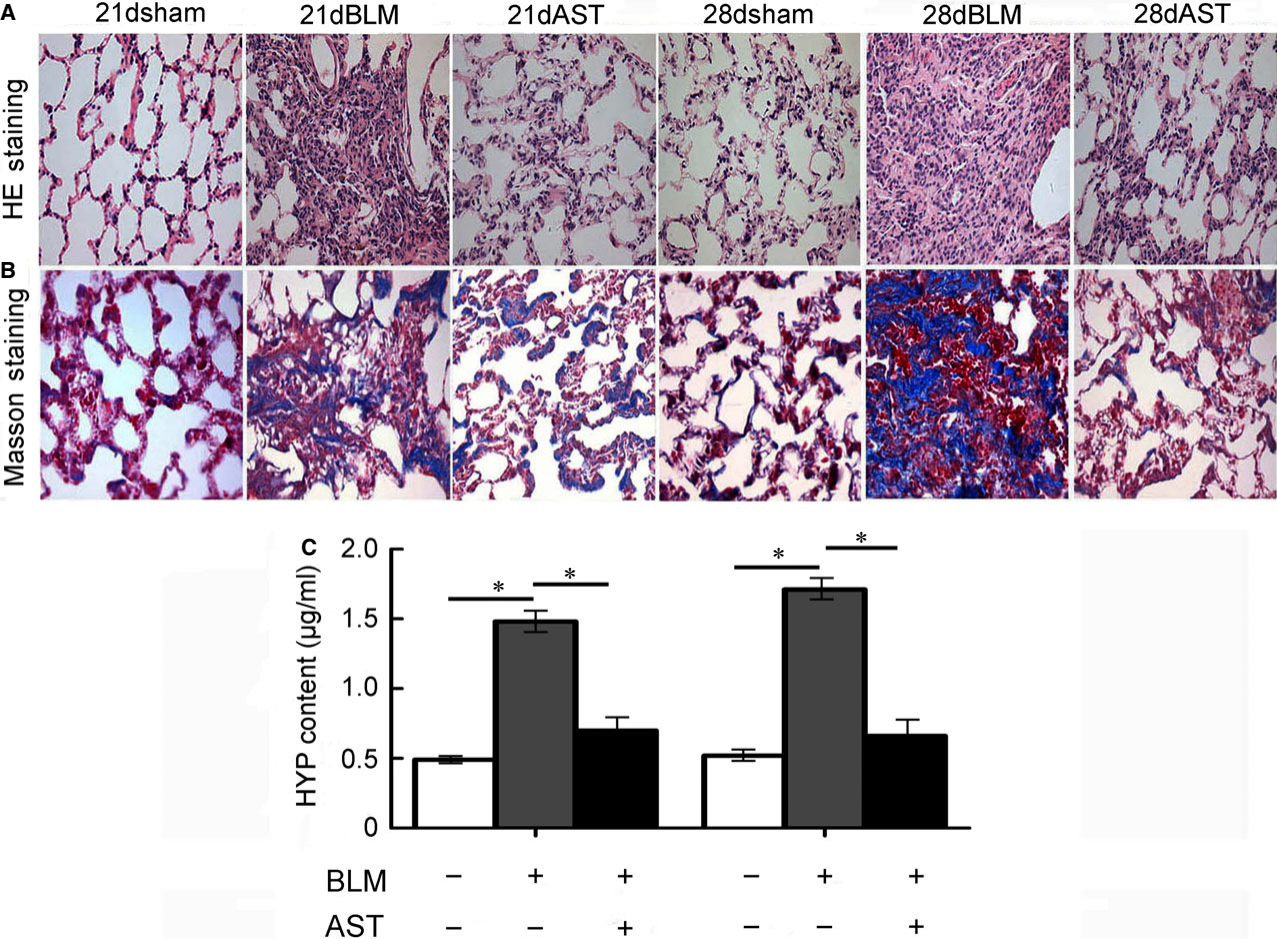
Prevention of astaxanthin on pulmonary fibrosis. (A) Representative images of haematoxylin and eosin stained lung sections from rats. (B) Lung tissue sections were stained using Masson’s trichrome for collagen (blue staining). (C) The content of hydroxyproline was significantly reduced by astaxanthin treated for 7 and 14 days. Data are shown as mean ± SD, *n* = 7, *P < 0.05 *versus* control group or BLM group, measured by Student–Newman–Keuls test. AST: astaxanthin, BLM: bleomycin.

**Figure 4:**
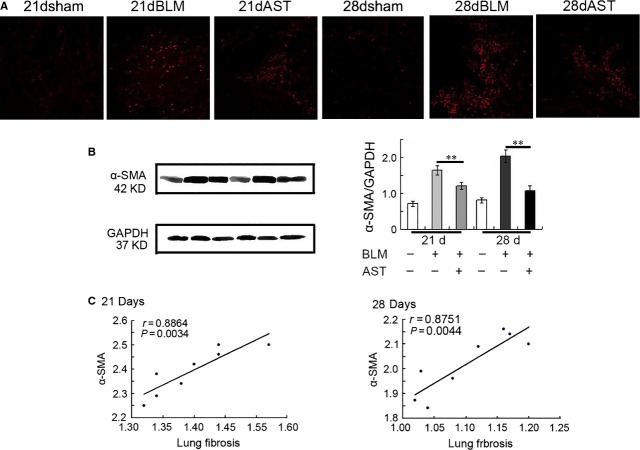
Astaxanthin reduced the number of myofibroblasts *in vivo*. (A) Astaxanthin reduced the number of myofibroblasts labelled with anti-α-SMA antibody. (B) The protein level of α-SMA was also reduced when detected by immunoblot assays. (C) Correlation between α-SMA-positive cells and fibrosis scoring. Correlation analysis was performed by spearman’s correlation coefficient. Data are shown as mean ± SD, *n* = 7, **P < 0.01 *versus* BLM group, measured by Student–Newman–Keuls test. AST: astaxanthin, BLM: bleomycin.

To detect the therapeutic effect of astaxanthin on lung fibrosis, we administered bleomycin-injured rats with or without astaxanthin in their post-inflammatory fibrotic phase. Bleomycin-induced fibrotic rats displayed increased lung parenchymal distortion, showing thicker alveolar membrane and severe oedema (Fig.3A). However, astaxanthin treatment evidently ameliorated these injuries. Astaxanthin markedly attenuated collagen deposition as shown by Masson’s trichrome staining (Fig.3B). An evident decrease in hydroxyproline was observed upon astaxanthin treatment for 7 and 14 days (Fig.3C). Myofibroblast is the most contributing cell for collagen. In this study, we assessed whether astaxanthin can reduce the number of myofibroblasts. The results showed that α-SMA-positive cells were even less in the lung tissues of astaxanthin-treated rats (Fig.4A). Moreover, astaxanthin treatment for 7 and 14 days significantly decreased the α-SMA protein level, which was consistent with the scoring of fibrosis (Fig.4B and C). These results indicated that astaxanthin ameliorated lung fibrosis partly because of its ability to reduce myofibroblasts in lung tissues.

### Astaxanthin promoted myofibroblast apoptosis

**Figure 5:**
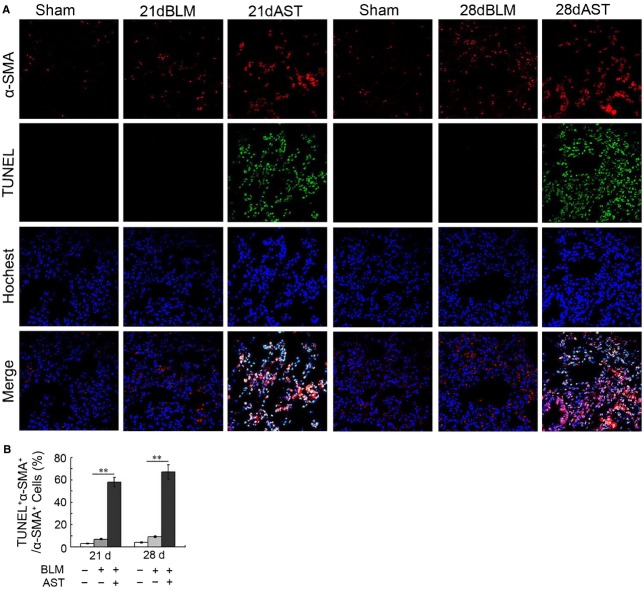
Astaxanthin promoted myofibroblasts apoptosis *in vivo*. (A) Representative fluorescent images of myofibroblast apoptosis in pulmonary sections examined by TUNEL assay (green). To identify the myofibroblasts, we stained pulmonary sections with anti-α-SMA antibody (red). (B) The number of dual-positive cells relative to α-SMA-positive cells was significantly increased in the lung tissue after astaxanthin treatment, especially in the day 28 images. At least 500 α-SMA^+^ cells per slide were counted. Data are shown as mean ± SD, *n* = 7, **P < 0.01 *versus* BLM group, measured by Student–Newman–Keuls test. AST: astaxanthin, BLM: bleomycin.

**Figure 6:**
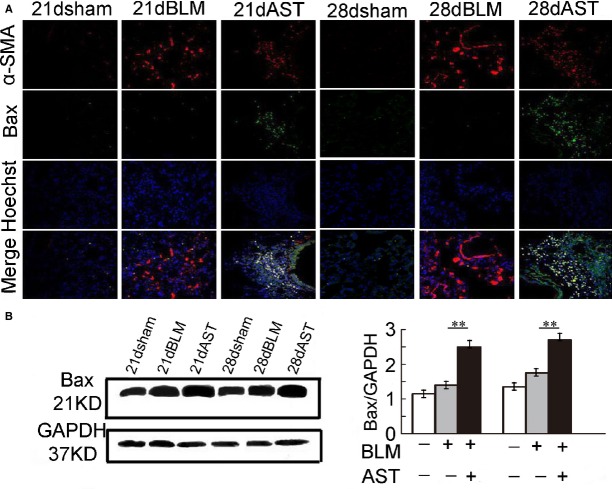
Astaxanthin promoted Bax expression in myofibroblast. (A) Representative fluorescent images of lung tissues labelled with the anti-α-SMA and anti-Bax antibodies. Yellow fluorescence represents fluorescence co-localization. Dual staining α-SMA^+^ Bax^+^ cells (yellow) were present in the lung tissue after astaxanthin treatment. (B) Immunoblot assays showed that astaxanthin increased the protein level of Bax. Data are shown as mean ± SD, *n* = 7, **P < 0.01 *versus* BLM group, measured by Student–Newman–Keuls test. AST: astaxanthin, BLM: bleomycin.

To further explore the effect of astaxanthin on myofibroblasts, we compared the myofibroblast apoptosis between the astaxanthin-treated and -untreated lung tissues after bleomycin instillation. Representative immunofluorescence images clearly showed that the number of TUNEL-positive cells significantly increased in the lung tissues of the astaxanthin-treated rats for 7 and 14 days compared with the astaxanthin-untreated rats after bleomycin exposure. Double immunofluorescence staining for TUNEL and α-SMA of lung tissues showed co-localization of TUNEL-positive and α-SMA-positive cells, indicating that TUNEL-positive cells were indeed myofibroblasts and astaxanthin treatment significantly promoted myofibroblast apoptosis (Fig.5A and B). Double immunofluorescence staining also revealed Bax overexpression induced by astaxanthin, which primarily occurred in myofibroblasts (Fig.6A). Western blot confirmed that Bax expression was highly up-regulated in astaxanthin-treated rats (Fig.6B).

**Figure 7:**
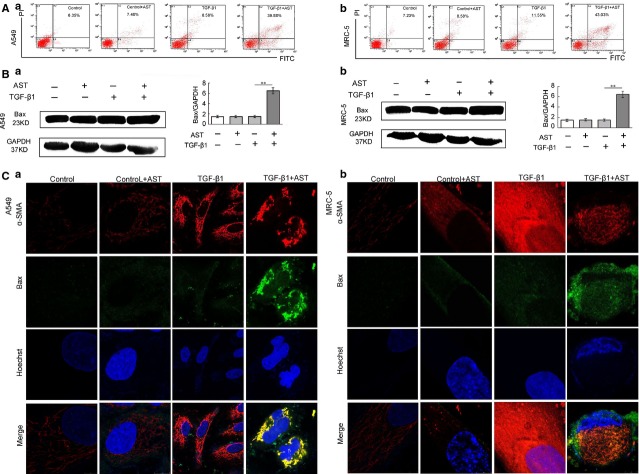
Astaxanthin promoted myofibroblasts apoptosis *in vitro*. A549 and MRC-5 cells pre-treated with TGF-β1 for 72 hrs and then co-treated with or without astaxanthin for 48 hrs. (A) Astaxanthin increased the rate of myofibroblast apoptosis determined by Annexin V-PI assay. (B) Immunoblot analysis showed elevation of Bax in astaxanthin-treated cells, as compared with the controls. (C) Increased cell apoptosis was also detected by dual fluorescence staining of α-smooth muscle actin (α-SMA) (red) and Bax (green). Yellow fluorescence represents fluorescence co-localization, which indicates myofibroblast (α-SMA^+^) apoptosis. Data are shown as mean ± SD, *n* = 6, **P < 0.01 *versus* TGF-β1 group, measured by Student–Newman–Keuls test. AST: astaxanthin.

Flow cytometry analysis showed that the apoptotic rate of cells increased from 8.58% representing the basal levels of apoptosis to 39.88% in TGF-β1-activated A549 cells and 11.55% representing the basal levels of apoptosis to 43.03% in TGF-β1-activated MRC-5 cells treated with astaxanthin for 48 hrs (Fig.7A). Western blot analysis also showed that astaxanthin increased Bax expression in TGF-β1-mediated A549 and MRC-5 cells (Fig.7B). Moreover, double immunofluorescence staining revealed that astaxanthin treatment significantly induced the Bax overexpression in myofibroblasts, although fibroblasts were insensitive to the apoptosis-inducing effects of astaxanthin (Fig.7C).

The *in vitro* and *in vivo* results strongly supported that astaxanthin can promote myofibroblast apoptosis.

### Astaxanthin-induced mitochondrial fission

**Figure 8:**
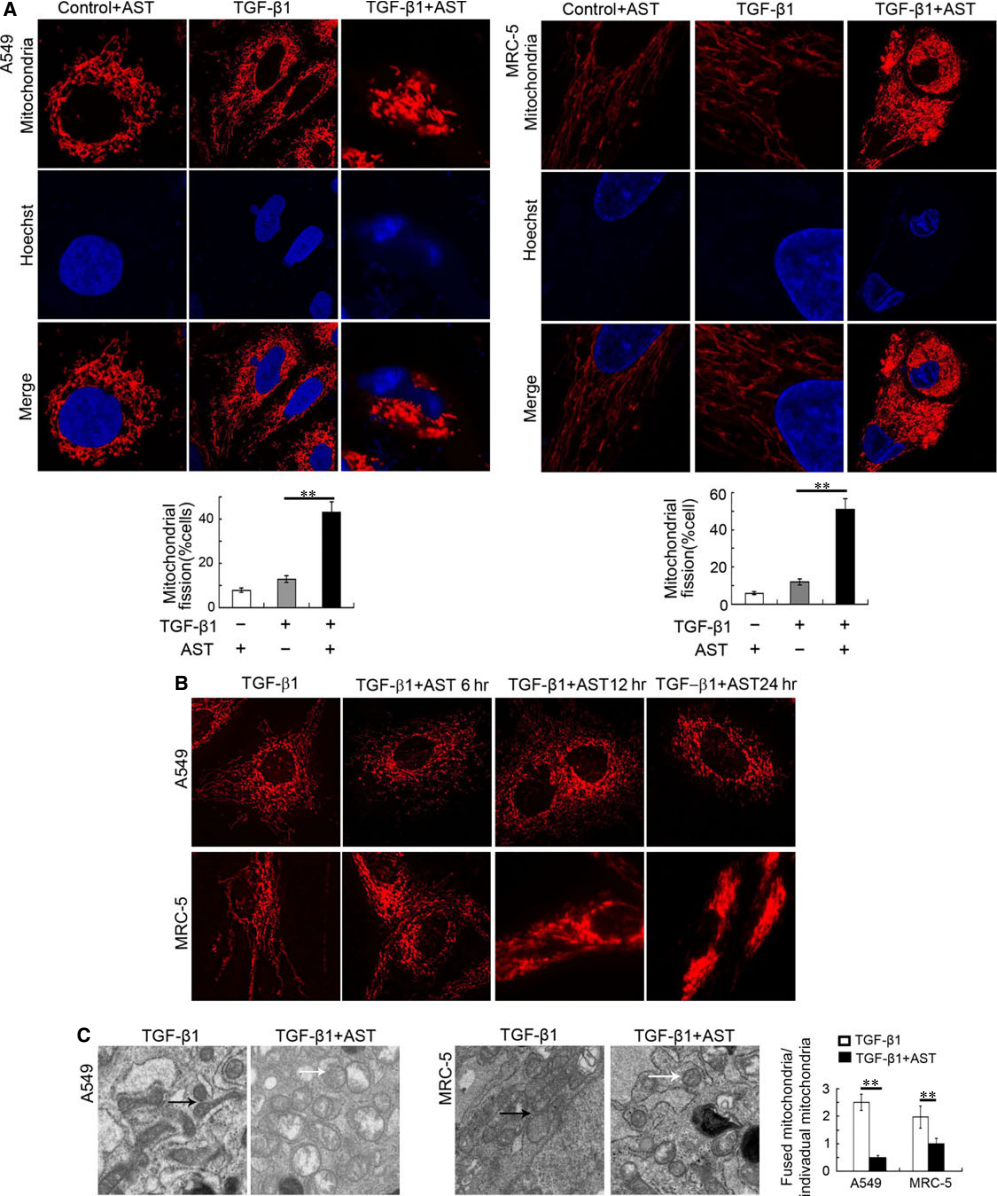
Astaxanthin promoted mitochondrial fission. A549 and MRC-5 cells were pre-treated with TGF-β1 for 72 hrs and subsequently co-treated with or without astaxanthin for 48 hrs. (A) Alterations of mitochondrial structures were analysed by a mitochondria-specific fluorescence probe, MitoTracker Red CMXRos. Representative photos showed mitochondrial fission induced by astaxanthin. The percentage of cells undergoing fission relative to the total number of cells were counted and summarized. Astaxanthin significantly increased the number of cells undergoing mitochondrial fission. At least 500 cells per parallel hole were counted. (B) Representative individual mitochondria were tracked in real-time using a live cell imaging system immediately after cells were exposed to astaxanthin. (C) Electron micrographs of mitochondria in A549 and MRC-5 cells. Black arrows point to the typical fused mitochondria, white arrows point to the typical fissional mitochondria (punctiform mitochondria). Quantitative analysis of ultrastructural changes was performed by averaging the ratio of fused mitochondria/fissional mitochondria for each experimental group. A total of 30 cells were scored in each parallel hole. More fissional mitochondria existed when cells were treated with astaxanthin. Data are shown as mean ± SD, *n* = 6, **P < 0.01 *versus* TGF-β1 group, measured by Student–Newman–Keuls test (A) or unpaired Student’s *t*-tests (C). AST: Astaxanthin

Increasing evidence has shown that mitochondrial fission plays a critical role in initiating cell apoptosis 22,23. We explored the regulatory effect of astaxanthin on mitochondrial fission. Confocal microscope images showed that the mitochondria of control myofibroblasts existed in a normal elongated tubular structure. However, astaxanthin caused nearly a fourfold increase in the number of cells displaying punctuated mitochondrial structures in TGF-β1-activated A549 and MRC-5 cells (Fig.8A). To further confirm this morphological change in the mitochondria, cells were tracked real-time by using the live cell imaging system immediately after astaxanthin treatment. The results showed that the tubular network of the mitochondria began to transform into fragmented independent organelles within 6 hrs after treatment (Fig.8B). Ultrastructural findings also showed that astaxanthin induced the mitochondria to transform from a long and filamentous structure into a round and short-shaped formation, which indicated mitochondrial fission. Quantitative results showed that astaxanthin significantly decreased the ratio of fused mitochondria/fissional mitochondria in the TGF-β1-activated A549 and MRC-5 cells (Fig.8C). These data indicated that astaxanthin can modulate mitochondrial fission.

### Drp1 is necessary for astaxanthin-induced mitochondrial fission and apoptosis

**Figure 9:**
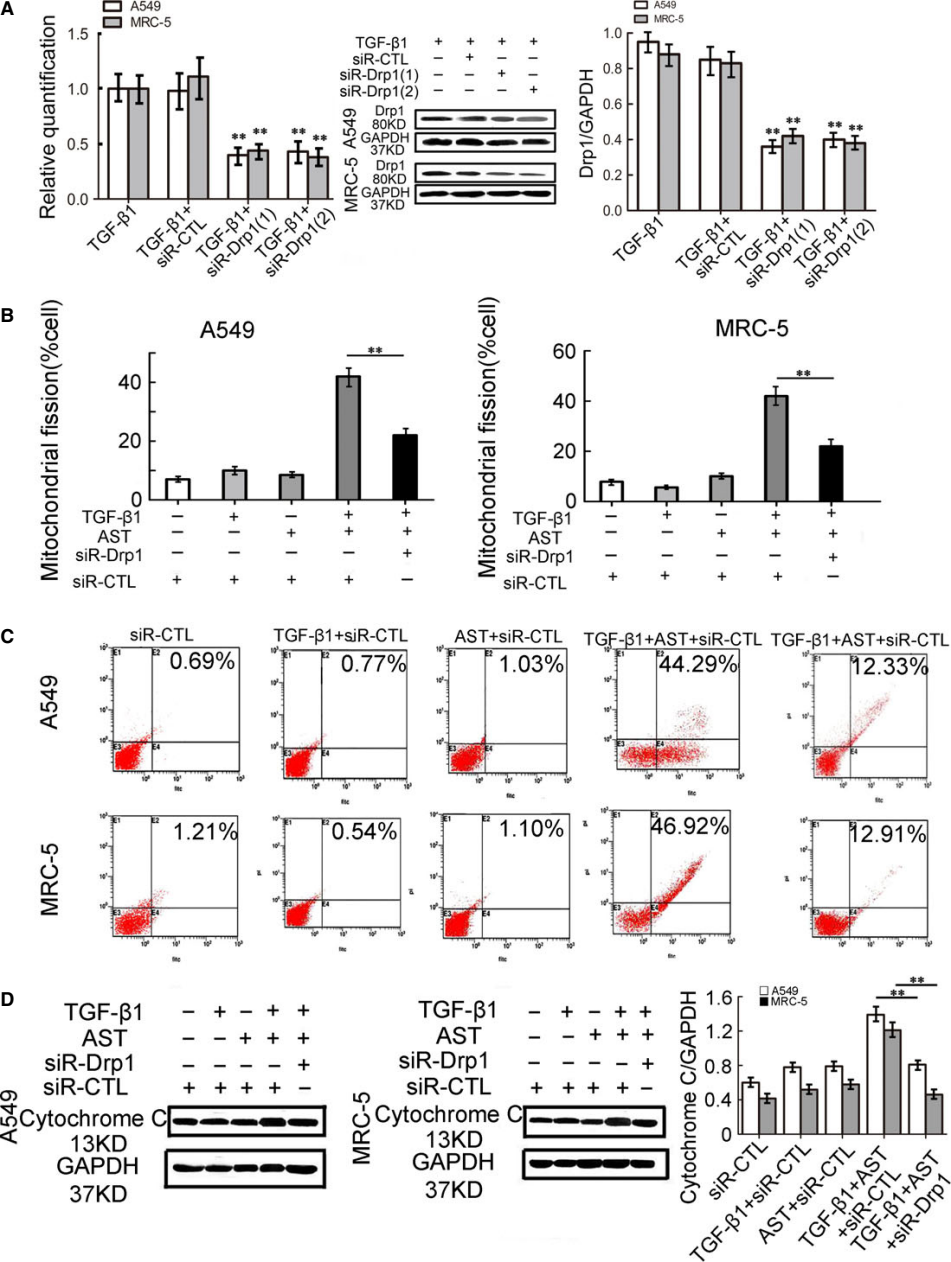
Astaxanthin promoted mitochondrial fission dependent on Drp1. (A) Alteration of DRP1 mRNA and protein levels in A549 and MRC-5 cell after knockdown. (B) The histogram showed the number of cells undergoing mitochondrial fission. Drp1 knockdown prevented astaxanthin-induced mitochondrial fission. (C and D) Function of Drp1 siRNA on astaxanthin-induced myofibroblast apoptosis was detected by flow cytometry and levels of cytochrome c. The rate of apoptosis was obviously reduced in Drp1 siRNA^+^ cells after astaxanthin treatment. The Drp1 siRNA^+^ group showed the least level of cytochrome c when treated with astaxanthin compared with others. Data are shown as mean ± SD, *n* = 6, **P < 0.01 *versus* TGF-β1 + siR-CTL group or TGF-β1 + AST+siR-CTL group, measured by Student–Newman–Keuls test. AST: astaxanthin.

Dynamin-related protein-1 is considered the key player in mitochondrial fission. We investigated whether the apoptotic response of myofibroblasts to astaxanthin was mediated by Drp1-dependent mitochondrial fission. Dynamin-related protein-1-specific siRNA was used to knockdown Drp1 in both TGF-β1-activated-A549 and MRC-5 cells. The efficiency of siRNA was measured by RT-PCR and Western blot analysis before and after transfection (Fig.9A). Considering the different efficiencies of two Drp1 siRNAs, activated-A549 cells were transfected with siR-Drp1 (1), whereas activated-MRC-5 cells were transfected with siR-Drp1 (2) for further studies. Two Drp1 siRNAs significantly reduced the number of cells undergoing mitochondrial fission caused by astaxanthin (Fig.9B). The siR-Drp1 (1) caused a 3.6-fold decrease in astaxanthin-induced cell apoptosis in activated-A549. The siR-Drp1 (2) caused nearly the same degree of reduction in astaxanthin-induced cell apoptosis in the activated-MRC-5 cells (Fig.9C). Western blot results showed that astaxanthin increased the level of cytoplasmic cytochrome c in the activated-A549 and MRC-5 cells, whereas the cytochrome c release induced by astaxanthin was largely abolished by Drp1 siRNA (Fig.9D). The results confirmed that astaxanthin promoted myofibroblast apoptosis mainly by activating Drp1-dependent mitochondrial fission pathway.

### Astaxanthin promoted Drp1 expression and translocation

**Figure 10:**
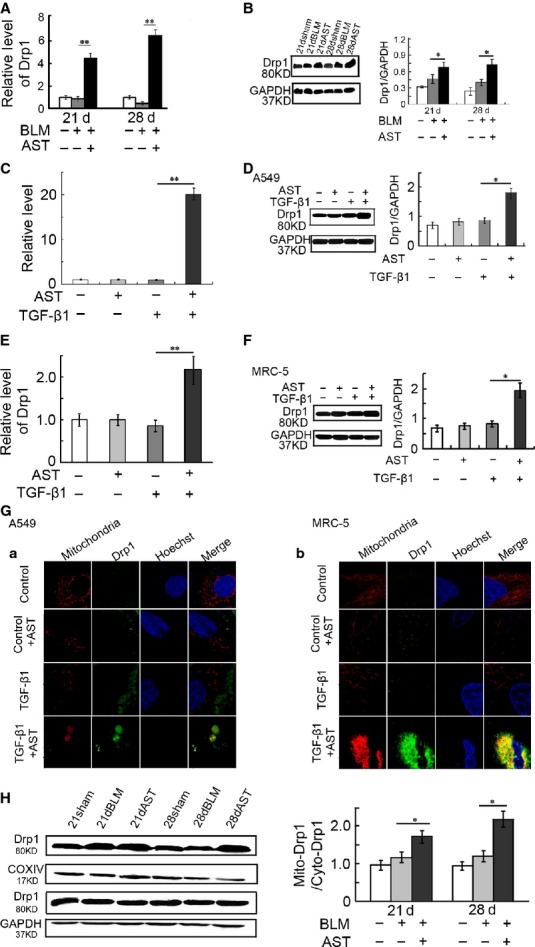
Astaxanthin promoted Drp1 expression and mitochondrial recruitment. A549 and MRC-5 cells were pre-treated with TGF-β1 for 72 hrs and subsequently co-treated with or without astaxanthin for 48 hrs. (A and B) Drp1 RNA and protein levels were detected by PCR and Western blot analysis in lung tissues. (C–F) Drp1 RNA and protein levels in A549 and MRC-5 cells. (G) Confocal images to detect fluorescence immunostaining of MitoTracker Red CMXRos and Drp1 (green) in A549 (A) and MRC-5 cells (B). Yellow fluorescence represents fluorescence co-localization, indicating that Drp1 was recruited to mitochondria after cells were exposed to astaxanthin. (H) Representative immunoblot of the protein levels of Drp1 in mitochondria and cytoplasm isolated from rat lung tissue. The quantification analysis is also presented. Data are shown as mean ± SD, *n* = 7, *P < 0.05, **P < 0.01 *versus* BLM group (A, B, H) or TGF-β1 group (C–F), measured by Student–Newman–Keuls test. AST: astaxanthin, BLM: bleomycin.

To understand the modulation of astaxanthin on Drp1, we detected the changes in expression and translocation of Drp1 in myofibroblasts with or without astaxanthin treatment. The results showed that the mRNA levels of Drp1 were significantly increased by 4.3- and 17.3-fold, respectively, in the lung tissues treated with astaxanthin for 7 and 14 days as compared with the bleomycin group (Fig.10A). The protein levels also increased (Fig.10B). Astaxanthin treatment significantly increased the Drp1 expression in mRNA (Fig.10C and E) and the protein levels (Fig.10D and F) in the TGF-β1-mediated A549 and MRC-5 cells, which further confirmed the *in vivo* findings.

Double immunofluorescence staining results showed that astaxanthin significantly promoted the accumulation of Drp1 in the mitochondria of the TGF-β1-activated A549 and MRC-5 cells (Fig.10G). Moreover, *in vivo* immunoblot analysis revealed that the mitochondrial Drp1 protein levels were significantly increased in the lung tissues treated with astaxanthin for 7 and 14 days compared with the bleomycin group (Fig.10H). These results suggested that astaxanthin can promote the translocation of Drp1 from cytosol to mitochondria *in vivo* and *in vitro*.

We also explored the Drp1-associated genes during mitochondrial fission. The results revealed that astaxanthin up-regulated the levels of p53, which yielded and elevated Drp1 levels *in vivo* (Fig.1A). Consistent with the change in p53, the level of PUMA also increased and correlated with the accumulation of Drp1 in the mitochondria induced by astaxanthin (Fig.1B).

**Figure 11:**
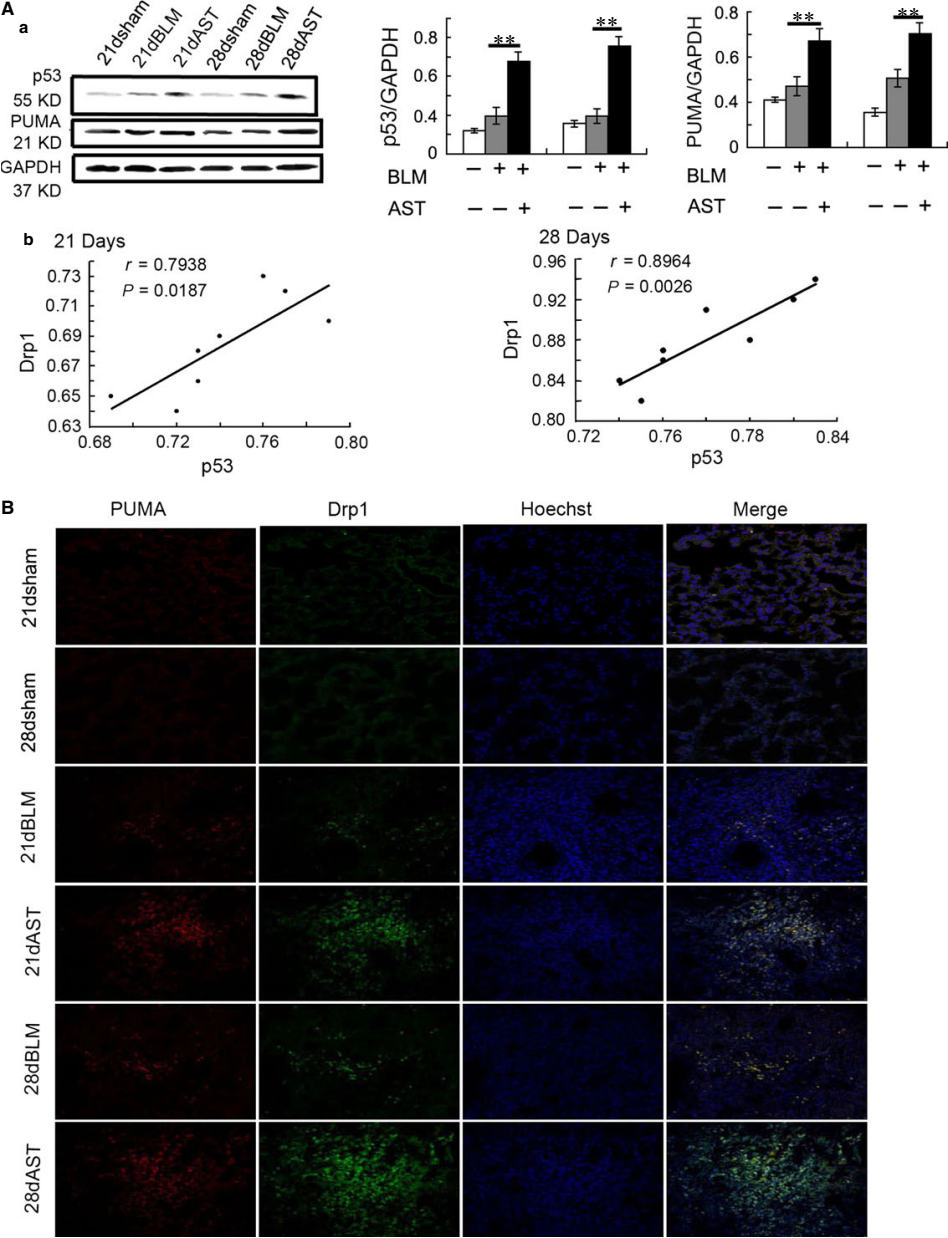
Astaxanthin enhanced Drp1-associated genes p53 and PUMA expression. (A) Western blot analysis showed p53 and PUMA expression were up-regulated in lung tissue after astaxanthin treatment (A). The correlations between p53 and Drp1 expression. Correlation analysis was performed by pearson correlation coefficient respectively (B). (B) Confocal images showed the distribution of Drp1 (green) and PUMA (red) in lung tissue. Yellow fluorescence represents fluorescence co-localization after astaxanthin treatment. Data are shown as mean ± SD, *n* = 6, **P < 0.01 *versus* BLM group, measured by Student–Newman–Keuls test. AST: astaxanthin, BLM: bleomycin.

## Discussion

Myofibroblasts are clinically and biologically important pathological features of pulmonary fibrosis, and their numbers on surgical lung biopsy directly correlate with progressive physiological deterioration and shortened survival 24,25. Although resident fibroblasts activate into myofibroblasts in response to injury, their origin in lung fibrosis remains controversial 26–28. Willis *et al*. 29 showed that AECs gradually lose their epithelial cell markers and polarity during human lung fibrogenesis. Simultaneously, these cells express mesenchymal markers and acquire single-cell motility. Kim *et al*. 30 also demonstrated the existence of epithelial–mesenchymal transition in lung biopsy samples from fibrotic patients. However, Yamada *et al*. 31 obtained conflicting results and did not detect double-positive cells for E-cadherin or surfactant-associated protein A, as well as α-SMA or vimentin, in lung tissues from patients with idiopathic pulmonary fibrosis and non-specific interstitial pneumonia. Furthermore, Rock *et al*. excluded pericytes and two epithelial cell populations as the origin of myofibroblasts 32. Our current work revealed that myofibroblasts, which can originate from AECs-II were detected in patients with pulmonary fibrosis. This phenomenon also existed in the lung tissues from bleomycin-induced lung fibrotic rats and cultured A549 and MRC-5 cells activated by TGF-β1. Thus, the use of these models is significant to further investigate the action of astaxanthin against pulmonary fibrosis by promoting myofibroblast apoptosis, which is based on Drp1-mediated mitochondrial fission.

A more recent study found that pulmonary fibrosis exhibited several cancer-like pathogenic features; thus, pulmonary fibrosis is considered to be a neoproliferative disorder of the lung 33. Similar to cancer cells, myofibroblasts show epigenetic and genetic abnormalities, as well as functional features such as uncontrolled proliferation, resistance to apoptosis and high migration rates 34. Thus, promotion of myofibroblast apoptosis is becoming a potential therapeutic strategy for fibrotic diseases 19. The current results showed that astaxanthin strongly improved lung parenchymal injury, reduced collagen deposition and promoted myofibroblast apoptosis through Drp1-mediated mitochondrial fission signalling pathway.

Several studies reported that astaxanthin effectively co-localized with mitochondria in many types of cells and tissues, such as leucocytes, mesangial cells, blastocysts, liver and muscles, implicating a role of astaxanthin in the mitochondria 35. Studies have shown that astaxanthin can impair the mitochondrial function and induce apoptosis in certain cancer cells through the mitochondrion-dependent pathways 36–38. Mitochondrial fission is important to maintain cellular function and initiate intrinsic apoptosis 39,40. Few functional food agents were identified to influence mitochondrial morphology leading to apoptosis. The present results showed that astaxanthin strongly promoted mitochondrial fission in myofibroblasts.

Dynamin-related protein-1, a conserved evolution protein, is one of the proteins proposed to participate in fission process 41. Dynamin-related protein-1 is a cytosolic protein with an N-terminal GTPase domain, a dynamin-like middle domain and a C-terminal GTPase effector domain, and considered as the principal activator of mitochondrial fission in a GTP-dependent manner 42,43. Mitochondrial fission may be controlled by regulating the expression and altering the activity of fission proteins, whereas Drp1 activity is often altered by mitochondrial recruitment, post-translational modification and conversion into its oligomer state 44. Its localization to the mitochondrial surface is important for mitochondrial fission 45. Zhang *et al*. 46 reported that overexpression of Drp1 was critical for cytoplasmic irradiation induced mitochondrial fission. Consistent with the previous work, our observation revealed that astaxanthin significantly increased Drp1 level and promoted the recruitment of Drp1 into mitochondria. Moreover, Drp1 deficiency by RNA interference and the use of a dominant-negative mutant protein (K38A) and a chemical inhibitor (mdivi-1) are known to delay the release of cytochrome c and subsequent apoptosis on challenge with apoptotic stimuli in various types of cultured mammalian cells 47,48. Din *et al*. 49 reported that Drp1 persistent accumulation in the mitochondria was associated with cell apoptosis or death. In agreement, Drp1 siRNA verified that Drp1 was directly essential for astaxanthin-induced mitochondrial fission leading to myofibroblast apoptosis.

Wang *et al*. 50 suggested that p53 presented direct implication for the regulation of mitochondrial fission through Drp1. Li *et al*. 51 also reported that Drp1 was transcriptionally up-regulated by p53, which conveyed the apoptotic signal of p53 by triggering mitochondrial fission in cardiomyocytes. The current data demonstrated that the regulation of astaxanthin on the Drp1 level was highly correlated with that of p53. PUMA is exclusively located in the mitochondria, which can facilitate the irreversible immobilization of Drp1 to the mitochondrial outer membrane through protein–protein interactions which form multimeric complexes 52,53. In agreement, the present data showed that Drp1 was co-localized with PUMA during astaxanthin-induced mitochondrial fission, leading to myofibroblast apoptosis.

In summary, we clarified the effects of pharmacological inhibition of astaxanthin on pulmonary fibrosis and found that this action required induction of myofibroblast apoptosis by activating the Drp1-mediated mitochondrial fission pathway. This finding suggested that Drp1 exhibits potential benefits for developing astaxanthin as a novel drug for pulmonary fibrosis.
